# Combined Treatment of Type 2 Diabetes and Hypothyroidism: Impact of Oral Semaglutide and Levothyroxine on Cardiometabolic and Thyroid Parameters: A 6-Month Comparative Study

**DOI:** 10.3390/epidemiologia7020041

**Published:** 2026-03-04

**Authors:** Dana-Mihaela Tilici, Ruxandra-Mihaela Costinescu, Diana Loreta Paun, Daniela Stegaru, Beatrice Mihaela Grecu, Mirona Costea, Cristian Guja

**Affiliations:** 1 Doctoral School (IOSUD) of “Carol Davila”, University of Medicine and Pharmacy, Dionisie Lupu 37, 030167 Bucharest, Romania; mirona.costea@drd.umfcd.ro; 2 Endocrinology Department, Bucharest University Emergency Hospital, Splaiul Independenței 169, 050098 Bucharest, Romania; diana.paun@umfcd.ro (D.L.P.); beatrice-mihaela.grecu@rez.umfcd.ro (B.M.G.); 3 Department of Endocrinology and Diabetology of “Carol Davila”, University of Medicine and Pharmacy, Eroii Sanitari 8, 050474 Bucharest, Romania; daniela.stegaru@umfcd.ro (D.S.); cristian.guja@umfcd.ro (C.G.)

**Keywords:** type 2 diabetes, hypothyroidism, oral semaglutide, levothyroxine, lipid profile, HbA1c, body mass index, GLP-1 receptor agonist

## Abstract

Background/Objectives: Type 2 diabetes (T2DM) and hypothyroidism often coexist, worsening cardiometabolic risk. Oral semaglutide and levothyroxine each improve metabolic parameters, but the effect of combined therapy is understudied. This study aimed to evaluate whether oral semaglutide administered concomitant with levothyroxine provides additive benefits on lipid profile, glycemic control, and body weight in patients with both conditions. Methods: This prospective comparative observational study assessed a total of 210 patients who were enrolled (70 per group) with a 6-month follow-up. Group A (T2DM and hypothyroidism) received semaglutide and levothyroxine, group B (hypothyroidism only) received levothyroxine, and group C (T2DM only) received oral semaglutide. Lipid profile, glycemic profile (HbA1c), thyroid profile, and anthropometric parameters were comparable across groups at baseline and after 6 months. Results: Group A demonstrated significant improvements in lipid parameters: LDL-cholesterol decreased by 12.7%, HDL increased by 9.0%, and triglycerides decreased by 6.7% (all comparisons *p* < 0.001 unless otherwise specified). In contrast, group B experienced worsening lipid profiles (LDL increased by 11.0%, HDL decreased by 0.5%, and triglycerides increased by 9.1%), while group C showed modest changes (LDL increased by 4.5%). Glycemic control improved among diabetic patients, with HbA1c declining by 7.7% in group A and 12.6% in group C. Body mass index (BMI) decreased in groups A (4.9%) and C (6.0%). Conclusions: The concurrent administration of oral semaglutide and levothyroxine produces additive cardiometabolic advantages in individuals with T2DM and hypothyroidism. These findings suggest that combined treatment may optimize metabolic outcomes in this particularly high-risk population.

## 1. Introduction

Type 2 diabetes mellitus (T2DM) and thyroid dysfunction (TD), particularly hypothyroidism, often coexist—a comorbidity pattern that raises significant clinical and therapeutic difficulties [1,2,3,4,5,6,7].

Compared to the general population, individuals with T2DM have a higher incidence of thyroid issues, as demonstrated by multiple epidemiological studies and meta-analyses [1,2,3,4,8]. Prevalence estimates can differ among regions and populations, indicative of geographic and demographic variations.

Recently, a meta-analysis of data from 2000 to 2022 reported a pooled prevalence of TD of about 20.2% in people with T2DM (95% CI: 17.9–22.6%), with many displaying subclinical or overt hypothyroidism [1]. Additionally, a descriptive study published in 2024 found that 36.8% of T2DM patients had thyroid disorders, with around 25% havingovert hypothyroidism [4].

The pathophysiological link between T2DM and TD is multifactorial and complex. Glycemic imbalanceshave beenproposed to impactthyroid hormone (TH) metabolism atseverallevels, includingat the hypothalamic-pituitary axis. These can also affect peripheral conversion of T4 to T3,which may result insubclinical or overt hypothyroidism [2,3,4,8,9]. Older age, female sex, longer diabetes duration, poor glycemic control (high HbA1c), central obesity, and diabetic complications (such as nephropathy and retinopathy) have also been identified as risk factors for coexisting TD [6,7,10,11,12,13]. The simultaneous presence of these two hormone disorders may exacerbate metabolic and cardiovascular risks from a clinical standpoint.

For example, a cross-sectional study conducted in India reported that hypothyroidism, overt or subclinical, in T2DM patients was independently associated with significantly higher odds of microvascular (retinopathy, nephropathy, neuropathy) and macrovascular complications (peripheral arterial disease), compared with euthyroid diabetics [4]. Thus, neglecting thyroid function in diabetic patients may compromise comprehensive risk stratification and management.

Conversely, recent advancementsin thepharmacological management ofT2DM, notably the introduction of glucagon-like peptide-1 receptor agonists (GLP-1 RAs) such as semaglutide, open upnewtherapeutic options [14]. Semaglutide has demonstrated the ability to enhance glycemic control, support weight loss, and positively affect various cardiometabolic risk factors (such as lipid levels and blood pressure) in addition to its glucose-lowering effects [15,16,17,18,19,20]. Specifically, in real-world observational studies, oral semaglutide use over 9 months was associated with significant reductions in HbA1c, BMI, and improved metabolic parameters among T2DM patients [21].

Despite the notable prevalence of TD in T2DM, there is a surprising lack of research on the combined management of T2DM and hypothyroidism using GLP-1 RAs together with TH replacement, such as levothyroxine. Additionally, the potential interactions between GLP-1 RA therapy and the thyroid axis are not well understood. In particular, prospective comparative data simultaneously addressing cardiometabolic outcomes, levothyroxine dose dynamics, thyroid morphology, and calcitonin behavior in patients receiving oral semaglutide are extremely limited. A comprehensive meta-analysis of randomized controlled trials assessing the safety of GLP-1 RAs found a slight increase in the overall likelihood of thyroid disorders (RR 1.28, 95% CI 1.03–1.60). Still, it did not show a significantly increased risk for specific conditions like overt hypothyroidism, hyperthyroidism, thyroiditis, goiter, or thyroid cancer [22].

Moreover, in a prospective study of obese T2DM patients treated with another GLP-1 RA (in this case, Exenatide), a significant reduction in serum thyroid stimulating hormone (TSH) was observed over 12 months, which correlated with weight loss, while free T4 levels remained unchanged [23].

These observations raise significant questions about the impact of GLP-1-driven weight loss and metabolic changes on thyroid function and hormone requirements.

Collectively, these findings highlight a critical gap in the evidence: what is the effect of combined treatment with GLP-1 RAs and levothyroxine on lipid metabolism, blood sugar regulation, weight, and TSH levels in individuals with both T2DM and hypothyroidism?

Investigating this matter is clinically relevant, as enhanced glycemic and lipid control may reduce cardiovascular risk; however, either insufficient or excessive treatment for thyroid issues could worsen metabolic homeostasis or cause adverse effects.

We hypothesized that concomitant administration of oral semaglutide and levothyroxine would result in additive or synergistic improvements in cardiometabolic parameters, while maintaining functional and structural thyroid stability.

Oral semaglutide was chosen because it shares similar pharmacokinetics and bioavailability with levothyroxine sodium, an aspect that is essential for the correlations examined in this study. Also, it represents a more recently available formulation that has been less extensively studied compared to the injectable form.

Therefore, in the present study, we systematically evaluated three well-defined patient groups over 6 months: (1) patients with T2DM and hypothyroidism treated with both oral semaglutideand levothyroxine (study group), (2) hypothyroid patients on levothyroxine only, and (3) T2DM patients treated with semaglutide only—allowing a rigorous comparison of combined therapy versus monotherapy in terms of lipid profile (LDL, HDL, triglycerides), glycemic control (fasting glucose, HbA1c), and an anthropometric parameter—body mass index (BMI). We aim to elucidate whether the dual therapeutic approach exerts additive or synergistic benefits, or whether there exist trade-offs in terms of metabolic or endocrine stability. Primary endpoints were changes in LDL, HDL, triglycerides, HbA1c, and BMI; secondary endpoints included TSH, fT4, fT3, levothyroxine dose variation, and calcitonin levels.

In doing so, we offer new insights into this clinical association within a relatively unexplored field that can help improve patient care in routine practice, especially given the increasing use of GLP-1 RAs and the high incidence of TD, particularly hypothyroidism, in T2DM.

## 2. Materials and Methods

A prospective clinical study was conducted to evaluate 6-month changes in key metabolic factors among patients receiving concomitant oral Semaglutide and Levothyroxine sodium. The study included patients who presented to both the Prof. Dr. N.C. Paulescu, National Institute of Diabetes, Nutrition and Metabolic Diseases, and the Bucharest University Emergency Hospital, and were recruited consecutively between June 2024 and September 2025. Although recruitment extended until September 2025, the present analysis includes only patients who completed the 6-month follow-up by the predefined data cutoff. Participants were selected during the study period according to established inclusion and exclusion criteria.

Data from three patient groups were analyzed, each comprising 70 participants aged 18 years or older. The reference group (study group A) consisted of patients with concomitant hypothyroidism (of any cause) and T2DM, treated with oral Levothyroxine sodium and Semaglutide. The two control groups included patients with hypothyroidism (of any cause) treated with Levothyroxine (control group B) and patients with T2DM treated with Semaglutide (control group C). A sample size of 70 participants per group was selected based on practical considerations of feasibility and patient availability during the recruitment period. For the reference group, the inclusion and exclusion criteria were as shown in Table 1. The control groups included patients with hypothyroidism who received only levothyroxine treatment and patients with T2DM who received only oral semaglutide treatment. The exclusion criteria remained unchanged.

In group B, Levothyroxine sodium was administered in the morning on an empty stomach, at least 30 min before a meal. In the reference group, Levothyroxine was given at least 30 min after oral Semaglutide administration, following current clinical recommendations. The Levothyroxine doses ranged from 25 to 150 µg per day in both groups. No medications known to interfere with Levothyroxine absorption were administered. The Semaglutide dose was standardized at 14 mg per day in both groups, and participants received it in the morning on an empty stomach. No additional antidiabetic medications were provided.

For each group, demographic data (sex, age, baseline BMI, and BMI at 6 months) were collected. Additionally, lipid profile values (LDL-cholesterol, HDL-cholesterol, triglycerides) and glucose profile values (fasting blood glucose, HbA1c) were measured at baseline and at 6 months. It is important to highlight that HbA1c was also measured in group B to ensure methodological consistency and to assess whether thyroid hormone replacement influences long-term glycemic markers in non-diabetic individuals.

For the main group and control group B, thyroid hormone profiles (TSH, fT4), daily Levothyroxine doses, and primary causes of hypothyroidism were also recorded. For the main group, dynamic measurements of Calcitonin were collected. Although fT3 was originally intended to be part of the hormonal assessment, inconsistent collection of fT3 values during follow-up led to their exclusion from the final analysis. In accordance with routine clinical practice, thyroid hormone parameters were not assessed in group C, as it comprised patients with type 2 diabetes mellitus without known thyroid disorders or indications for thyroid function monitoring.

Because of the observational study design and the characteristics of the interventions, investigators were not blinded to group allocation. All biochemical measurements, however, were performed using standardized laboratory assays independent of clinical grouping.

Statistical analyses included only complete-case data. Participants with missing baseline or follow-up values for a parameter were excluded from analyses of that parameter. Normality for each variable was assessed using the Shapiro–Wilk test to determine the appropriate statistical test (parametric test for normal distribution, respectively, non-parametric test for non-normal distribution). Statistical analysis was performed in two stages. First, changes in the specified parameters within each group at baseline and at 6 months were analyzed using statistical tests such as the Student t-test and the Wilcoxon signed-rank test. Data from 2 groups were also analyzed using an independent Student t-test or Mann–Whitney test, depending on the data distribution. Second, parameter values were compared over time across all three groups, with the main group (A) serving as the reference. Comparisons of categorical variables between groups were conducted using the χ^2^ (chi-square) test. For data that were not normally distributed, the Kruskal–Wallis test was used, followed by Dunn’s post hoc comparisons. Multiple linear regression analyses were performed. The multivariable linear regression models included treatment group, age, and baseline BMI as covariates. Results are presented as regression coefficients (B), 95% confidence intervals, and *p*-values. No formal adjustment for multiple comparisons was applied, as the analyses were predefined and hypothesisdriven. Therefore, *p*-values should be interpreted as exploratory. JASP Software (version 0.19.3, JASP Team, Amsterdam, The Netherlands) was used for statistical analysis, with significance set at *p* < 0.05 (two-tailed).

## 3. Results

### 3.1. Demographic and Clinical Characteristics

Each patient group was analyzed separately at first. More than half of the individuals in most groups were between 45 and 64 years old, except in group A, where this age range made up 48.6%. The majority of participants in each group were women, with a maximum of 91.4% in group B. A percentage of 55.2% of all patients in the study had hypertension (116/210). At baseline, 52.8% of patients were classified as obese (111/210), and most of these individuals were diagnosed with both T2DM and hypothyroidism. These patients made up about 79% of group C. Baseline comparisons revealed statistically significant differences between groups in age, sex distribution, and BMI (all *p* < 0.05). Table 2 provides a more detailed presentation of these data.

### 3.2. Distribution of Key Metabolic Parameters Within Groups

When analyzed separately, patients in group A showed a significant improvement in their lipid profile. LDL cholesterol decreased by approximately 12.7% over 6 months. HDL cholesterol also increased by about 9%, and triglycerides decreased by roughly 6.7%. In contrast, the same trends were not observed in the control groups. In group B, LDL cholesterol increased by approximately 11%, HDL cholesterol decreased by 0.5%, and triglycerides increased by 9.1%. Meanwhile, in group C, LDL cholesterol increased by 4.5%, while HDL cholesterol rose by 4.4%, and triglycerides showed a minimal decrease of 0.7%.

Additionally, patients with both T2DM and hypothyroidism experienced a smaller reduction in HbA1c (7.7%) compared to those with only T2DM, who saw a 12.6% decrease. The group without diabetes mellitus showed a slight increase of about 1.2%.

Lastly, body mass index decreased by 6% in group C and by 4.9% in group A. Patients in group B experienced a 4% weight increase from their initial value six months ago.

The statistical significance of these data is summarized in Table 3.

The primary metabolic parameters were analyzed between groups, revealing significant differences in absolute changes from baseline (all *p* < 0.05). These data are detailed in Table 4.

### 3.3. The Main Causes of Hypothyroidism

Autoimmune etiology was the predominant cause of hypothyroidism, accounting for 70.0% of cases in group A and 58.6% in group B. Within group A, 20.4% (10/49) of patients with autoimmune hypothyroidism presented with the atrophic form, while 61.2% (30/49) exhibited ultrasound features consistent with the goitrous form. In addition, central hypothyroidism, a rare cause of hypothyroidism, defined as hypothalamic or pituitary deficiency, diagnosed by inappropriately low or normal TSH levels in the presence of reduced circulating free thyroid hormone concentrations, was identified in 8.6% of patients in group B. Postprocedural hypothyroidism was observed in both groups. The distribution of hypothyroidism etiologies and their relative frequencies are summarized in Table 5.

### 3.4. Lipid Profile Variations

Multiple linear regression analysis showed that both monotherapies were significantly less effective in reducing LDL-cholesterol compared with the combined treatment. Control group B demonstrated an average reduction in LDL-cholesterol levels that was 23.26 mg/dL smaller (B = 23.26, 95% CI [14.16, 32.36], *p* < 0.001), while control group C showed a 26.04 mg/dL smaller reduction (B = 26.04, 95% CI [16.45, 35.62], *p* < 0.001). Age (*p* = 0.92), sex (*p* = 0.2), and BMI (*p* = 0.44) were not significant predictors of LDL-cholesterol reduction. Overall, the model explained 18.8% of the variance in LDL-cholesterol change (R^2^ = 0.188, *p* < 0.001), indicating that treatment was the primary determinant of LDL-cholesterol reduction.

Similar trends were observed in triglyceride levels over 6 months since both monotherapies were significantly less effective at improving levels. The control group B exhibited a 17.62 mg/dL smaller reduction (B = 17.62, 95% CI [8.09, 27.15], *p* < 0.001), while group C showed an 8.24 mg/dL smaller reduction (B = 8.24, 95% CI [−1.79, 18.26], *p* = 0.10). Although the regression model demonstrated overall statistical significance (*p* < 0.001), it accounted for only a modest proportion of the variance in triglyceride change (R^2^ = 0.10). Additionally, neither age (*p* = 0.07), sex (*p* = 0.6), nor BMI (*p* = 0.55) was a significant predictor of triglyceride reduction. These results suggest that treatment was the principal determinant of triglyceride response.

In our study, the model did not explain the variability in HDL-cholesterol levels over time (R^2^ = 0.02, adjusted R^2^ = 0.004). Neither age (*p* = 0.89), sex (*p* = 0.4), BMI (*p* = 0.79), nor treatment showed a statistically significant effect. These results indicate that the variables included in the model explained only a small proportion of HDL-cholesterol variation during the 6-month follow-up period. This outcome implies that HDL-cholesterol dynamics in this cohort are likely influenced primarily by unmeasured factors, including baseline HDL levels, lifestyle behaviors, or genetic predisposition, rather than by age, BMI, or treatment allocation.

Although the results are statistically significant, the R^2^ values suggest that additional factors, not captured by the regression models, contribute to the observed outcomes.

### 3.5. BMI Variations

Regarding the change in BMI, the Kruskal–Wallis test showed significant differences between treatments (*p* < 0.001). Post hoc analyses established that group C had the greatest reduction (vs. group A, *p* < 0.005; vs. group B, *p* < 0.001), followed by group A (vs. group B, *p* < 0.001).

### 3.6. Glycated Hemoglobin Variations

Diabetes control was best evaluated by measuring HbA1c. In our study, the HbA1c variation over 6 months was significant in all three groups (*p* < 0.001). In group B (hypothyroidism without T2DM), although changes in HbA1c achieved statistical significance, the values remained within the non-diabetic range and were thus considered exploratory rather than clinically meaningful. The largest reduction was seen in group C, which showed a decrease of 1.06% more than group B (*p* < 0.001). Group A displayed an intermediate reduction, 0.69% higher than control group B (*p* < 0.001), but 0.37% lower than control group C (*p* < 0.001).

### 3.7. Thyroid Hormone Profile

Thyroid hormone profile values for groups A and B were collected at baseline and after 6 months. The reference group exhibited more stable TSH variation than group B. In group A, median TSH levels decreased from 3.1 mUI/L [2.1–4.7] at baseline to 2.6 mUI/L [1.9–3.3] at 6 months (*p* < 0.001; large effect size: r = 0.61). In group B, although some individual cases showed notable percentage changes due to low baseline values, these changes were not statistically significant. Median TSH levels in group B were 1.86 mUI/L [0.7–3.6] at baseline and 1.82 mUI/L [0.85–2.9] at 6 months (*p* = 0.15). The absence of statistical significance is likely due to substantial interindividual variability in TSH changes, as indicated by the wide dispersion and overlapping distributions at both time points, resulting in a small effect size (r = 0.20).

**Figure 1:**
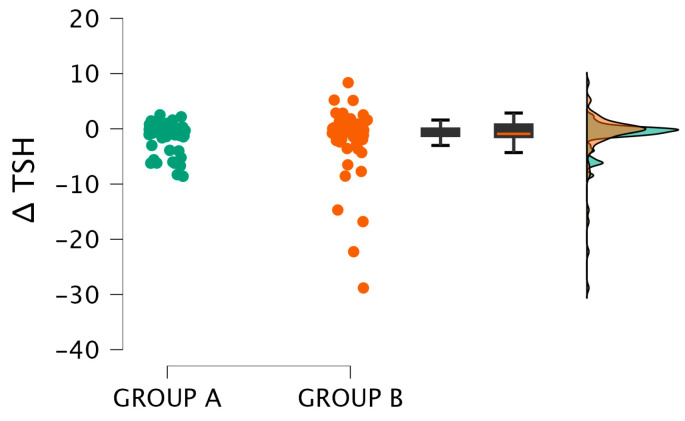
The distribution of changes in TSH levels (ΔTSH) in Groups A and B is presented. Individual data points are displayed alongside boxplots that indicate the median and interquartile range. The groups exhibit substantial overlap, consistent with the non-significant Mann–Whitney U test result.

Changes in TSH values were also compared between the two groups. Although group A showed a substantial decrease in TSH levels, the difference between the two groups was not statistically significant (*p* = 0.104; r = 0.16). Figure 1 presents a graphical representation of the data.

Serum fT4 values were analyzed longitudinally in both groups. In group A, where fT4 values were non-normally distributed, no statistically significant change was observed, with median fT4 increasing from 1.05 ng/dL [0.9–1.6] at baseline to 1.2 ng/dL [1.01–1.45] at follow-up (*p* = 0.07; r = 0.25). In group B, fT4 values were normally distributed and remained stable over time, with mean values of 0.91 ng/dL ± 0.2 SD at baseline and 0.90 ng/dL ± 0.16 SD at six months (*p* = 0.8).

Thyroid hormone measurements were not routinely collected in group C because these patients had no documented thyroid disease and were not undergoing thyroid-related treatment.

### 3.8. Variation in Levothyroxine Doses

A key part of our study was to analyze how Levothyroxine doses change over six months in group A and the control group B, while considering variation in TSH levels (R^2^ = 0.185). Compared to group A, patients in group B showed an average increase of 5.46 mcg in the Levothyroxine dose (B = 5.46, 95% CI [0.72, 10.19], *p* = 0.02), indicating they require larger and more variable dose adjustments. Conversely, patients in group A needed significantly smaller changes in Levothyroxine dose.

The decreased need for levothyroxine dose adjustments seen in the combination therapy group indicates greater thyroid functional stability over the six-month follow-up, possibly reflecting a more predictable hormonal response compared to levothyroxine monotherapy.

### 3.9. Dynamics of Calcitonin Values

A particularly important aspect of our study was examining changes in calcitonin levels within our group. Initially, the average was 3.4 pg/mL ± 3.8 SD. After 6 months, there was roughly an 11.4% increase, with the average rising to 4.1 pg/mL ± 4.77 SD (*p* < 0.001). However, the values remained within normal limits (<10 pg/mL), and the ultrasound appearance of the thyroid remained unchanged during the 6 months.

## 4. Discussion

The relationship between T2DM and thyroid disorders, particularly hypothyroidism, is well established. However, additional research is required, especially regarding the pharmacological treatments administered to this patient population [24].

Existing data indicate that patients with T2DM have a higher prevalence of thyroid disorders compared to the general population [1,2,3,4,8]. These findings underscore the importance of routinely assessing thyroid function in the management of T2DM, particularly in the context of newer antidiabetic therapies that may influence the hypothalamic–pituitary–thyroid axis.

The increasing use of glucagon-like peptide-1 (GLP-1) receptor agonists, such as semaglutide, has raised new questions regarding the safety and efficacy of combination therapy, particularly in patients with thyroid comorbidities. This study aims to address a significant gap: what impact does combining GLP-1 RA and levothyroxine have on lipid profiles, blood sugar control, weight, and hormonal balance—issues that directly influence treatment choices. Recent reviews indicate that the relationship between GLP-1 agonists and thyroid function remains ambiguous, with studies often reporting conflicting outcomes [1,22,23]. Capuccio et al. recently noted that, despite the increasing use of GLP-1 receptor agonists in T2DM and obesity, evidence regarding their impact on thyroid function remains inconsistent [22]. Our results contribute to this debate by providing real-world data suggesting clinically relevant metabolic benefits without overt short-term adverse thyroid effects.

The present study demonstrates that combination therapy with oral semaglutide and levothyroxine leads to significant improvements in lipid profiles (a decrease in LDL of approximately 12.7% and an increase in HDL of approximately 9%), moderate improvement in glycemic control (a reduction in HbA1c of 7.7%), and a reduction in BMI (4.9%). These findings are consistent with recent clinical and observational studies indicating that semaglutide enhances glycemic control, promotes weight loss, and exerts favorable cardiometabolic effects, including improvements in lipid profiles, in both diabetic and obese populations [25,26,27,28]. Importantly, our data suggest that these benefits are maintained even in the presence of treated hypothyroidism, and that the inclusion of levothyroxine for thyroid management may exert a synergistic effect on lipid metabolism, explaining the magnitude of the observed benefits.

Insufficiently compensated hypothyroidism is associated with reduced LDL receptor expression, impaired hepatic lipid clearance, and decreased lipoprotein lipase activity, mechanisms that may explain the observed increases in LDL-cholesterol and triglycerides despite levothyroxine therapy, particularly in the absence of concomitant weight loss or insulin-sensitizing treatment [29]. The metabolic improvements observed in the combination group support the hypothesis that GLP-1 RA–induced weight loss and insulin sensitization may partially offset these adverse mechanisms.

The glycemic response was more pronounced in the semaglutide monotherapy group (group C), likely due to greater weight loss and the potential effect of thyroid dysfunction on insulin sensitivity. Previous studies indicate that compensated hypothyroidism can impact both glycemic regulation and the response to GLP-1 receptor agonists, suggesting that baseline patient characteristics contribute to the observed differences between groups [22,30]. In contrast, HbA1c values in the hypothyroidism-only group (group B) remained stable and within the non-diabetic range throughout the follow-up period. This finding supports the interpretation that the reductions observed in groups A and C were attributable to treatment effects rather than to background variability in glycemic markers.

The slightly greater reduction in HbA1c observed in patients treated exclusively with oral semaglutide may suggest a potential interaction affecting drug absorption when levothyroxine is co-administered. However, this interpretation is speculative and should be considered hypothesis-generating, as it is derived from indirect clinical observations and requires confirmation in dedicated pharmacokinetic and pharmacodynamic studies.

### 4.1. Semaglutide-Thyroid Metabolic Interaction and Implications for Levothyroxine Dosing

A reduced need for levothyroxine dose adjustments in the combination group compared with those receiving levothyroxine alone is a significant finding of this study. This result aligns with reports in the “gray” literature and clinical studies indicating that GLP-1 receptor agonists (GLP-1 RAs) can affect the thyroid axis, particularly by lowering TSH levels [22,31,32]. Recent research has demonstrated TSH reductions with both exenatide and semaglutide, an effect likely mediated by weight loss and central changes in TSH secretion [23,31,32].

These findings have two important clinical implications. Since levothyroxine replacement therapy is typically titrated based on body weight (expressed in µg/kg/day), weight loss resulting from GLP-1 receptor agonist therapy can modify the pharmacokinetic and pharmacodynamic requirements for thyroid hormone replacement [33,34]. Consequently, patients undergoing significant GLP-1 RA-associated weight loss demand systematic reevaluation and potential downward adjustment of their levothyroxine dosage to ensure maintenance of euthyroid status and to prevent overtreatment.

Conversely, several pathophysiological mechanisms may act in the opposite direction, leading to reduced effectiveness of levothyroxine therapy through changes in peripheral T4→T3 conversion or tissue sensitivity to thyroid hormones. These shifts may rarely require higher doses in some patients, especially those with fluctuating metabolic states or altered deiodinase activity. Moreover, semaglutide’s ability to delay gastric emptying adds another layer of complexity, since levothyroxine’s absorption varies widely and depends on bioavailability. Decreased gastric motility, changes in intragastric dissolution, and extended exposure to fluctuating pH levels can disrupt or unpredictably alter levothyroxine absorption.

Given these considerations, careful attention to the timing of administration is crucial to reduce drug interactions and maintain stable thyroid hormone function. Current guidelines recommend that oral semaglutide should be taken first, on an empty stomach, followed by levothyroxine at least 30 min later. This staggered dosing helps minimize competitive absorption, avoids the effects of semaglutide-induced gastric stasis, and maintains the pharmacokinetic integrity and therapeutic effectiveness of both medications.

In the context of this study, these findings highlight several clinically relevant considerations when combining levothyroxine with GLP-1 receptor agonist therapy. Weight loss-related changes in thyroid hormone requirements, potential alterations in drug absorption due to delayed gastric emptying, and central effects on TSH secretion underscore the need for systematic reassessment of levothyroxine dosing after GLP-1 RA initiation. Careful timing of administration, with oral semaglutide taken first and levothyroxine administered at least 30 min later, appears essential to maintain stable thyroid function. Regular biochemical monitoring of thyroid parameters is therefore warranted to ensure sustained euthyroidism and to minimize the risk of under- or overtreatment in routine clinical practice.

### 4.2. Thyroid Morphological Changes and Calcitonin Monitoring

TSH is a thyroid-proliferative factor, so its reduction—frequently observed under GLP-1 RA—could have a protective effect on thyroid volume. However, the literature data are contradictory: some studies did not find significant changes in thyroid volume or nodularity despite a decrease in TSH [24,33,34,35].

In all our groups, the ultrasound reassessment at 6 months revealed no detectable structural changes in the thyroid gland. Patients who initially had thyroid nodules at baseline maintained consistent characteristics during follow-up, with no changes in size and no sonographic signs of progression. These results suggest that, during this period, neither the reduction in TSH levels nor the metabolic changes induced by their treatment had a significant effect on thyroid structure.

Experimental studies in animal models have suggested that the administration of GLP-1 receptor agonists may stimulate thyroid C-cell proliferation at high doses; however, the relevance of these findings to human pathology remains uncertain [36,37]. Over the past two decades, large observational analyses and population-based cohorts with long-term follow-up have not consistently demonstrated an increased incidence of thyroid cancer in users of GLP-1 Ras [36,37,38,39]. Although some data sets describe a diagnostic peak in the first year of treatment, this phenomenon is predominantly interpreted as detection bias, and consolidated data through 2025 provide increasing reassurance about absolute tumor risk. Overall, the literature supports a cautious attitude and appropriate monitoring, but does not identify convincing evidence for a net increased risk of thyroid carcinoma in humans [14,36,37,38,39,40,41,42,43,44].

Regarding the results of our study, calcitonin’s behavior supports a cautious interpretation. The average 11.4% increase in calcitonin at six months, without exceeding pathological limits or significant structural ultrasound changes, indicates that these biochemical fluctuations might precede morphological changes but do not directly signify tumor progression. Nonetheless, this trend underscores the importance of medium- and long-term monitoring, especially in patients with pre-existing risk factors.

Currently, there are no standardized guidelines for monitoring calcitonin levels in patients with T2DM treated with GLP-1 RAs. However, an increasing number of publications recommend including thyroid hormone evaluation in routine screening, particularly in cases involving combined levothyroxine therapy. Prospective studies spanning several years, focusing on patients with thyroid dysfunction who are undergoing both GLP-1 RAs and levothyroxine treatment, are needed to systematically assess hormonal marker changes, calcitonin dynamics, and thyroid morphological alterations. Additionally, careful risk stratification—including family or personal history of thyroid cancer, presence of suspicious nodules, autoimmune diseases, or previous drug treatments—could help tailor monitoring and therapeutic decisions in high-risk cases. It is important to consider all factors contributing to fluctuations in calcitonin levels, as common autoimmune conditions like autoimmune thyroiditis and frequently used medications such as antacids can cause non-pathological increases in calcitonin. Based on current evidence, our recommendation aligns with existing studies and recommends avoiding GLP-1 analogues in patients with a family history of MTC or personal suspicion of MTC.

Simultaneously, as supported by current practice and the study, pre-treatment calcitonin assessment may be considered as an expert opinion–driven approach rather than a recommendation supported by formal international guidelines, particularly in patients with additional risk factors for thyroid disease.

### 4.3. Limitations and Future Research Directions

This study has several limitations that warrant acknowledgment; however, these should be interpreted in the context of the study’s clearly defined exploratory aims. The observational, non-randomized design does not allow for causal inference, yet it is appropriate for addressing real-world clinical outcomes in a population that remains understudied in randomized trials. The six-month follow-up period, while relatively short, was sufficient to detect clinically eloquent changes in metabolic and thyroid-related parameters that are known to respond early to GLP-1 RAs therapy.

The predominance of female participants reflects the epidemiology of hypothyroidism in routine clinical practice and, while it may limit extrapolation to male populations, augments the internal validity of the findings for the population most frequently affected. Although the sample size controls statistical power for subgroup analyses and the detection of rare adverse events, it remains comparable to or larger than that of several previously published observational studies in this field, and it provides hypothesis-generating data that support the need for larger investigations.

The current literature remains fragmented, with most studies featuring short follow-up periods, diverse populations (including individuals with obesity, diabetes, and thyroid conditions), and considerable variability in the hormonal markers evaluated.

In most oral semaglutide safety studies, thyroid function is rarely taken into consideration, and is only evaluated as a secondary safety endpoint, which raises the risk of confounding and may lead to underreporting of clinically important changes [24]. Furthermore, baseline heterogeneity in thyroid disease causes and levothyroxine dosing may have influenced individual responses to treatment. In the absence of specific, evidence-based guidelines for patients with concomitant T2DM and thyroid disease, emerging literature increasingly advocates for the routine assessment of thyroid function in individuals treated with GLP-1 receptor agonists. The present study is fully aligned with this evolving clinical perspective and provides supportive real-world data reinforcing the rationale for systematic thyroid monitoring in this complex patient population.

Prospective studies are needed that specifically focus on patients with T2DM and hypothyroidism (both autoimmune and non-autoimmune) who are treated with GLP-1 RAs. Such studies should incorporate long-term monitoring of thyroid-stimulating TSH, fT4, calcitonin, and thyroid antibodies, as well as assessment of thyroid structural changes using ultrasound to evaluate volume and nodular progression. Randomized controlled designs and pharmacokinetic analyses would be particularly valuable to clarify potential interactions between GLP-1 RAs and levothyroxine absorption. Correlating these data with levothyroxine dosage, metabolic parameters, and weight loss patterns would enhance understanding of the underlying endocrine interactions. Furthermore, risk stratification for patients with a history of thyroid disease or oncological predisposition could facilitate the development of personalized treatment strategies. These approaches are essential for establishing effective monitoring protocols and for identifying patients most likely to benefit from combination therapy.

## 5. Conclusions

This study assessed whether the concomitant administration of oral semaglutide and levothyroxine confers additional benefits for lipid profile, glycemic control, and body weight in patients with both hypothyroidism and metabolic dysfunction. The results indicate that combination therapy generates significant metabolic improvements, consistent with the positive outcomes previously reported for injectable GLP-1 receptor agonists. Patients showed reductions in blood glucose and cholesterol levels without necessitating changes in levothyroxine dosage, as TSH responses remained stable during the six-month follow-up period.

Additionally, no significant alterations in thyroid morphology were observed during the six-month follow-up, as ultrasound reassessment revealed stable glandular structure and unchanged characteristics of pre-existing nodules across all groups. These findings support the short-term structural safety of concomitant oral semaglutide and levothyroxine therapy, despite the known reduction in TSH levels frequently associated with GLP-1 receptor agonist treatment.

Nonetheless, thyroid function can change even without dose adjustments, so ongoing monitoring remains crucial. Regular assessments should include thyroid ultrasound combined with biochemical tests of TSH, calcitonin, fT4, and preferably fT3, as this comprehensive approach allows for early detection of both functional and structural thyroid changes. Specifically, ultrasound surveillance is clinically important for identifying potential structural alterations that could be relevant for CMT risk, while calcitonin monitoring offers additional biochemical safety information.

These conclusions are based on a 6-month follow-up and are most applicable to similar real-world outpatient populations. In summary, these results imply that the concomitant use of oral semaglutide and levothyroxine is safe and may provide additive metabolic benefits, supporting its potential application in the comprehensive management of patients with hypothyroidism and metabolic disorders.

## Figures and Tables

**Table 1:** The inclusion and exclusion criteria for the participants.

Inclusion Criteria	Exclusion Criteria
Adults diagnosed with both T2DM and hypothyroidism	Diagnosis of type 1 diabetes mellitus
Concomitant treatment with oral semaglutide and levothyroxine	Age < 18 years
Provision of written informed consent	Known hypersensitivity to the active substance or any excipients
	History of gastric or bariatric surgery
	Contraindications of GLP-1 RA
	Absence of informed consent
	Incomplete follow-up data

**Table 2:** Demographic and anthropometric profile of the study population.

*n* (%)		Group A	Group B	Group C	Total (*n* = 210)	*p*-Value Between Groups
Age (years)	<45	0	11 (15.7%)	21 (30%)	32 (15.2%)	<0.001
	45–64	34 (48.6%)	40 (57.1%)	45 (64.3%)	119 (56.7)	
	≥65	36 (51.4%)	19 (21.1%)	4 (5.7%)	59 (28.1%)	
Sex	female	53 (75.7%)	64 (91.4%)	38 (54.3%)	155 (73.8%)	<0.001
	male	17 (24.3%)	6 (8.6%)	32 (45.7%)	55 (26.2%)	
BMI (kg/m^2^)	Underweight (<18.5 kg/m^2^)	0	1 (1.4%)	0	1 (0.5%)	<0.001
	Normal (18.5–24.9 kg/m^2)^	3 (4.3%)	15 (21.4%)	1 (1.4%)	19 (9%)	
	Overweight (25–29.9 kg/m^2^)	34 (48.6%)	31 (44.3%)	14 (20%)	79 (37.6%)	
	Obesity (≥30 kg/m^2^)	33 (47.1%)	23 (32.9%)	55 (78.6%)	111 (52.9%)	
Hypertension		53 (75.7%)	23 (32.8%)	40 (57.1%)	116 (55.2%)	

**Table 3:** Baseline and 6-month comparative analysis of metabolic parameters.

		Group A	Group B	Group C
LDL-cholesterol (mg/dL)	First presentation	129 ± 32.1 SD	112.1 ± 37.9 SD	156.6 ± 27.1 SD
	6 months-later	110.5 ± 25.6 SD	117.9 ± 36.8 SD	163.9 ± 21.9 SD
	*p*-value	<0.001	0.07	<0.001
HDL-cholesterol (mg/dL)	First presentation	43.9 ± 10 SD	60.2 ± 15.1 SD	43.3 ± 6.7 SD
	6 months-later	46.8 ± 8.3 SD	59.5 ± 16.4 SD	44.2 ± 11.3 SD
	*p*-value	<0.001	0.15	0.55
Triglyceride (mg/dL)	First presentation	163.3 ± 58.5 SD	106.4 ± 53 SD	179.8 ± 44.8 SD
	6 months-later	149.2 ± 42.4 SD	111.5 ± 59 SD	172.6 ± 44.6 SD
	*p*-value	<0.001	0.08	<0.001
HbA1c (%)	First presentation	8.0 ± 0.73 SD	5.5 ± 0.61 SD	7.7 ± 1.0 SD
	6 months-later	7.4 ± 0.69 SD	5.6 ±0.61 SD	6.7 ± 0.7 SD
	*p*-value	<0.001	0.003	<0.001
Fasting plasma glucose (mg/dL)	First presentation	134.5 ± 43.7 SD	90.6 ± 19.7 SD	157.4 ± 24.4 SD
	6 months-later	116.7 ± 34.4 SD	94.5 ± 21.6 SD	128.3 ± 23.5 SD
	*p*-value	<0.001	0.009	<0.001
BMI (kg/m^2^)	First presentation	30.9 ± 4.5 SD	28.0 ± 4.3 SD	32.1 ± 2.9 SD
	6 months-later	29.5 ± 4.0 SD	28.5 ± 4.1 SD	30.2 ± 2.8 SD
	*p*-value	<0.001	<0.001	<0.001

The values are the mean ± SD. LDL-cholesterol = low-density lipoprotein cholesterol; HDL-cholesterol = high-density lipoprotein cholesterol; HbA1c = hemoglobin A1c; BMI = body mass index.

**Table 4:** Comparison of 6-month changes in metabolic parameters between groups.

	Group A	Group B	Group C	*p*-Value Between Groups
Δ ^1^ LDL-cholesterol (mg/dL)	−18.5 ± 20.9 SD	5.83 ± 36.6 SD	7.33 ± 10.0 SD	<0.001
Δ HDL-cholesterol (mg/dL)	2.8 ± 6.42 SD	−0.77 ± 8.92 D	0.94 ± 13.3 SD	0.009
Δ Triglyceride (mg/dL)	−14.1 ± 26 SD	5.04 ± 37.3 SD	−1.2 ± 4.2 SD	<0.001
Δ HbA1c (%)	−0.62 ± 0.4 SD	0.06 ± 0.2 SD	−0.99 ± 0.5 SD	<0.001
Δ BMI (kg/m^2^)	−1.6 ± 1.4 SD	0.4 ± 5.8 SD	−1.96 ± 1.09 SD	<0.001

^1^ Δ = Absolute change from baseline to 6-month follow-up.

**Table 5:** Causes of hypothyroidism.

Hypothyroidism	Group A	Group B
Autoimmune	49 (70%)	41 (58.6%)
Postprocedural	21 (30%)	23 (32.8%)
Central	0	6 (8.6%)

## Data Availability

The original contributions presented in this study are included in the article. For further inquiries, please contact the corresponding authors.

## Abbreviations

The following abbreviations are used in this manuscript:

BMI	body mass index
fT4	free thyroxine
GLP-1 RAs	glucagon-like peptide-1 receptor agonists
HbA1c	hemoglobin A1c
HDL-c	high-density lipoprotein cholesterol
LDL-c	low-density lipoprotein cholesterol
MTC	Medullary thyroid cancer
T2DM	Type 2 diabetes mellitus
TD	thyroid dysfunction
TGL	triglycerides
TH	thyroid hormone
TSH	thyroid-stimulating hormone

## References

[1] Hadgu R., Worede A., Ambachew S. (2024). Prevalence of thyroid dysfunction and associated factors among adult type 2 diabetes mellitus patients, 2000–2022: A systematic review and meta-analysis. Syst. Rev..

[2] Grigoriadis G., Koufakis T., Kotsa K. (2023). Epidemiological, Pathophysiological, and Clinical Considerations on the Interplay between Thyroid Disorders and Type 2 Diabetes Mellitus. Medicina.

[3] Tilici D.-M., Paun D.L., Arnautu A.M., Mirica A., Duta C., Costea M., Guja C. (2025). The Intricate Relationship Between Thyroid Disorders and Type 2 Diabetes—A Narrative Review. Diabetology.

[4] Patel P.R., Maitra A., Ashok A., Jose J., Ragav Y., Paul N.N. (2025). Evaluation of Thyroid Dysfunction in Type 2 Diabetes Mellitus Patients and Its Association With Diabetic Complications: A Cross-Sectional Study. Cureus.

[5] Nishi M. (2018). Diabetes mellitus and thyroid diseases. Diabetol. Int..

[6] Catma Y., Edizer A., Bayramlar O.F., Gul N., Selcukbiricik O.S., Karsidag K., Uzum A.K. (2025). Higher Prevalence of Thyroid Dysfunction in Type 2 Diabetes Mellitus: Effects on Glycemic Control, Diabetic Complications and Comorbidities. Medicina.

[7] Elmenshawi I.M. (2017). Prevalence of Thyroid Dysfunction in Diabetic Patients. J. Diabetes Metab. Disord. Control.

[8] Kandel L., Shakya Y.L., Yadav M., Shah N.A., Gupta S. (2024). Prevalence of Thyroid Dysfunction among Patients with Type II diabetes Mellitus in Tertiary Care Center: A Cross-sectional Descriptive Study. JNMA J. Nepal. Med. Assoc..

[9] Azad A.R.A.J., Zohara Z. (2025). The Interplay Between Thyroid Disorders and Diabetes and Their Impact on Cardiovascular Outcomes: A Systematic Review. Cureus.

[10] Kalra S., Aggarwal S., Khandelwal D. (2019). Thyroid Dysfunction and Type 2 Diabetes Mellitus: Screening Strategies and Implications for Management. Diabetes Ther..

[11] Ogbonna S.U., Ezeani I.U. (2019). Risk factors of thyroid dysfunction in patients with type 2 diabetes mellitus. Front. Endocrinol..

[12] Biondi B., Kahaly G.J., Robertson R.P. (2018). Thyroid Dysfunction and Diabetes Mellitus: Two Closely Associated Disorders. Endocr. Rev..

[13] Haider M.Z., Rehman M.A.U., Mufti T.A., Anwar A., Ain Q.U., Rabbani R.A., Jamil M.I. (2025). Frequency and Clinical Correlates of Thyroid Dysfunction in Patients with Type 2 Diabetes Mellitus: A Cross-Sectional Study. Cureus.

[14] Zheng Z., Zong Y., Ma Y., Tian Y., Pang Y., Zhang C., Gao J. (2024). Glucagon-like peptide-1 receptor: Mechanisms and advances in therapy. Signal Transduct. Target. Ther..

[15] Okamoto A., Yokokawa H., Nagamine T., Fukuda H., Hisaoka T., Naito T. (2021). Efficacy and safety of semaglutide in glycemic control, body weight management, lipid profiles and other biomarkers among obese type 2 diabetes patients initiated or switched to semaglutide from other GLP-1 receptor agonists. J. Diabetes Metab. Disord..

[16] Seighali N., Gholami-Chahkand M.S., Ebrahimzade M., Hosseini K. (2025). Effect of Oral Semaglutide on Cardiometabolic Risk Factors in Overweight and Obese Individuals with or Without Diabetes: A Systematic Review and Meta-Analysis. https://ssrn.com/abstract=5803144.

[17] Candido R., Di Loreto C., Desenzani P., Pantanetti P., Romano C., Settembrini S., Fadini G.P. (2024). Suitability and Usefulness of a Flexible Dosing Timing of Oral Semaglutide to Maximize Benefit in Clinical Practice: An Expert Panel. Diabetes Ther..

[18] Gašparini D., Čakanić F., Tufekčić T., Rasnek D., Wensveen F.M., Turk Wensveen T. (2024). Oral semaglutide improves glycemic control and lowers blood lipids: Evidence from a real-world study. Cardiovasc. Endocrinol. Metab..

[19] MacIsaac R.J. (2025). Semaglutide: A key medication for managing cardiovascular-kidney-metabolic syndrome. Future Cardiol..

[20] Alkhatib M., Almasri N., Alshwayyat S., Almahariq H., Hammadeh B.M., Taimeh Z., Al-Kurdi M.A.M. (2025). The multifaceted effects of semaglutide: Exploring its broad therapeutic applications. Future Sci. OA.

[21] Aneja P., Bhalla G., Jain A. (2025). Safety and Efficacy of Semaglutide in Type 2 Diabetes Patients: A Real-World Study. Int. J. Diabetes Technol..

[22] Capuccio S., Scilletta S., La Rocca F., Miano N., Di Marco M., Bosco G., Di Pino A. (2024). Implications of GLP-1 Receptor Agonist on Thyroid Function: A Literature Review of Its Effects on Thyroid Volume, Risk of Cancer, Functionality and TSH Levels. Biomolecules.

[23] Tee S.A., Tsatlidis V., Razvi S. (2023). The GLP-1 receptor agonist exenatide reduces serum TSH by its effect on body weight in people with type 2 diabetes. Clin. Endocrinol..

[24] Hauge C., Breitschaft A., Hartoft-Nielsen M.L., Jensen S., Bækdal T.A. (2021). Effect of oral semaglutide on the pharmacokinetics of thyroxine after dosing of levothyroxine and the influence of co-administered tablets on the pharmacokinetics of oral semaglutide in healthy subjects: An open-label, one-sequence crossover, single-center, multiple-dose, two-part trial. Expert Opin. Drug Metab. Toxicol..

[25] Magomedova A. (2025). The Effects of Semaglutide in Adults (18+) with Overweight or Obesity and Diabetes type 2. A Narrative Review. Med. Res. Arch..

[26] Alarfaj S.J. (2025). The Effectiveness of Semaglutide on a Composite Endpoint of Glycemic Control and Weight Reduction and Its Effect on Lipid Profile Among Obese Type 2 Diabetes Patients. Medicina.

[27] Palazzi S., Sentinelli F., Zugaro A., Morgante S., Santarelli L., Melanzi S., Baroni M.G. (2025). Real-World Analysis of Short-Term Effectiveness of Oral Semaglutide: Impact on Glycometabolic Control and Cardiovascular Risk. Pharmaceuticals.

[28] Milushewa P., Mitreva Y., Chakarova N., Tankova T., Naseva E., Petkova V. (2025). Predictive factors for HbA1c and weight loss associated with semaglutide treatment in type 2 diabetes mellitus: Real-world clinical evidence. Front. Endocrinol..

[29] Alnahdi H.M. (2025). Exploring hypothyroidism’s effects on lipid profiles: Evidence of metabolic consequences in subclinical disease. Saudi Med. J..

[30] Hu W., Song R., Cheng R., Liu C., Guo R., Tang W., Liu J. (2022). Use of GLP-1 Receptor Agonists and Occurrence of Thyroid Disorders: A Meta-Analysis of Randomized Controlled Trials. Front. Endocrinol..

[31] Mazza A.D. (2025). The Thyroid Twist: How GLP-1 Agonists Are Influencing Autoimmune Thyroid Care. Cureus.

[32] Kupnicka P., Król M., Żychowska J., Łagowski R., Prajwos E., Surówka A., Chlubek D. (2024). GLP-1 Receptor Agonists: A Promising Therapy for Modern Lifestyle Diseases with Unforeseen Challenges. Pharmaceuticals.

[33] Reinehr T., Andler W. (2002). Thyroid hormones before and after weight loss in obesity. Arch. Dis. Child..

[34] Agnihothri R.V., Courville A.B., Linderman J.D., Smith S., Brychta R., Remaley A., Celi F.S. (2014). Moderate weight loss is sufficient to affect thyroid hormone homeostasis and inhibit its peripheral conversion. Thyroid.

[35] Patel S., Niazi S.K., Patel S., Niazi S.K. (2025). Emerging Frontiers in GLP-1 Therapeutics: A Comprehensive Evidence Base. Pharmaceutics.

[36] Hale P.M., Ali A.K., Buse J.B., McCullen M.K., Ross D.S., Sabol M.E., Tuttle R.M., Stemhagen A. (2020). Medullary Thyroid Carcinoma Surveillance Study: A Case-Series Registry. Thyroid.

[37] Lisco G., De Tullio A., Disoteo O., Piazzolla G., Guastamacchia E., Sabbà C., Triggiani V. (2023). Glucagon-like peptide 1 receptor agonists and thyroid cancer: Is it the time to be concerned?. Endocr. Connect.

[38] Brito J.P., Herrin J., Swarna K.S., Singh Ospina N.M., Montori V.M., Toro-Tobon D., McCoy R.G. (2025). GLP-1RA Use and Thyroid Cancer Risk. JAMA Otolaryngol. Head. Neck Surg..

[39] Morales D.R., Bu F., Viernes B., Duvall S.L., Matheny M.E., Simon K.R., Suchard M.A. (2025). Risk of Thyroid Tumors With GLP-1 Receptor Agonists: A Retrospective Cohort Study. Diabetes Care.

[40] Smits M.M., Van Raalte D.H. (2021). Safety of Semaglutide. Front. Endocrinol..

[41] Toft D.J. (2019). Glucagon-like Peptide 1 Receptor Agonists Do Not Alter Calcitonin Levels in Humans. Clin. Thyroid..

[42] Chiu W.Y., Shih S.R., Tseng C.H. (2012). A Review on the Association between Glucagon-Like Peptide-1 Receptor Agonists and Thyroid Cancer. J. Diabetes Res..

[43] Angelyn Bethel M., Patel R.A., Thompson V.P., Merrill P., Reed S.D., Li Y., Holman R.R. (2019). Changes in Serum Calcitonin Concentrations, Incidence of Medullary Thyroid Carcinoma, and Impact of Routine Calcitonin Concentration Monitoring in the EXenatide Study of Cardiovascular Event Lowering (EXSCEL). Diabetes Care.

[44] Hegedüs L., Moses A.C., Zdravkovic M., Le Thi T., Daniels G.H. (2011). GLP-1 and Calcitonin Concentration in Humans: Lack of Evidence of Calcitonin Release from Sequential Screening in over 5000 Subjects with Type 2 Diabetes or Nondiabetic Obese Subjects Treated with the Human GLP-1 Analog, Liraglutide. J. Clin. Endocrinol. Metab..

